# Endocannabinoid signaling in the lateral habenula regulates pain and alcohol consumption

**DOI:** 10.1038/s41398-021-01337-3

**Published:** 2021-04-14

**Authors:** Rao Fu, Ying Tang, Wenfu Li, Zhiheng Ren, Ding Li, Jiayi Zheng, Wanhong Zuo, Xuejun Chen, Qi Kang Zuo, Kelsey L. Tam, Yucong Zou, Thomas Bachmann, Alex Bekker, Jiang-Hong Ye

**Affiliations:** 1 Department of Anesthesiology, Pharmacology, Physiology & Neuroscience, Rutgers, The State University of New Jersey, New Jersey Medical School, Newark, NJ 07103 USA; 2 Department of Anatomy, School of Medicine, Sun Yat-sen University, Guangzhou, Guangdong 510080 China; 3 Department of Microbiology, Biochemistry and Molecular Genetics, Rutgers, The State University of New Jersey, New Jersey Medical School, Newark, NJ 07103 USA

**Keywords:** Addiction, Psychology

## Abstract

Hyperalgesia, which often occurs in people suffering from alcohol use disorder, may drive excessive drinking and relapse. Emerging evidence suggests that the lateral habenula (LHb) may play a significant role in this condition. Previous research suggests that endocannabinoid signaling (eCBs) is involved in drug addiction and pain, and that the LHb contains core components of the eCBs machinery. We report here our findings in rats subjected to chronic ethanol vapor exposure. We detected a substantial increase in endocannabinoid-related genes, including *Mgll* and *Daglb* mRNA levels, as well as monoacylglycerol lipase (MAGL) protein levels, as well as a decrease in *Cnr1* mRNA and type-1 cannabinoid receptor (CB1R) protein levels, in the LHb of ethanol-exposed rats. Also, rats withdrawing from ethanol exposure displayed hypersensitivity to mechanical and thermal nociceptive stimuli. Conversely, intra-LHb injection of the MAGL inhibitor JZL184, the fatty acid amide hydrolase inhibitor URB597, or the CB1R agonist WIN55,212-2 produced an analgesic effect, regardless of ethanol or air exposure history, implying that alcohol exposure does not change eCB pain responses. Intra-LHb infusion of the CB1R inverse agonist rimonabant eliminated the analgesic effect of these chemicals. Rimonabant alone elicited hyperalgesia in the air-, but not ethanol-exposed animals. Moreover, intra-LHb JZL184, URB597, or WIN55,212-2 reduced ethanol consumption in both homecages and operant chambers in rats exposed to ethanol vapor but not air. These findings suggest that LHb eCBs play a pivotal role in nociception and facilitating LHb eCBs may attenuate pain in drinkers.

## Introduction

Alcohol use disorders (AUD) affect 15 million Americans (Substance Abuse and Mental Health Services Administration, 2015) and are closely tied to chronic pain in humans. AUD and chronic pain share common neural circuits giving rise to the possibility that chronic pain states could significantly affect alcohol use patterns, and AUD could influence pain sensitivity^1^. Recent studies suggest that around 1 in 4 adults in chronic pain report self-medicating with alcohol, and 43–73% of people with AUD report experiencing chronic pain. An improved understanding of the effects of alcohol on pain and the role of pain in alcohol misuse will hopefully improve treatment outcomes for patients with such pain disorders.

Various brain circuits have been implicated in AUD-related pain, including central reward circuits involving the nucleus accumbens and medial prefrontal cortex (mPFC) networks, emotion circuits composed of the amygdala and the mPFC, as well as stress pathways^2^. The lateral habenula (LHb) has recently emerged as an essential brain region that controls the expression of aversive behaviors, including those related to alcohol^3–8^. Work from our laboratory has shown that the LHb plays an important role in anxiety- and depression-like behaviors associated with AUD^9–11^, and that these aberrant behaviors are concomitant with increased activity of, as well as glutamatergic transmissions to, LHb neurons^11,12^.

The endocannabinoid (eCB) system, which includes the neuronal type-1 cannabinoid receptor (CB1R) and the endogenous CB1R agonists 2-arachidonoyglycerol (2-AG) and N-arachidonylethanolamide (anandamide, AEA), plays a significant role in the etiology of drug addiction, among other physiological and pathological conditions^13,14^. A growing body of evidence shows that chronic alcohol exposure downregulates brain CB1R expression and function. A post-mortem study of alcohol-dependent humans reported disruptions of CB1R expression in the ventral striatum and cortical regions^15^, and in vivo imaging studies reported lower CB1R availability in heavy-drinking alcoholics^16,17^. Using an electron microscope, Berger et al.^18^ detected CB1Rs in the pre-and postsynaptic sites of both excitatory and inhibitory LHb neurons^18^. Research also shows that eCB signaling involves long-term depression (LTD) in the LHb^19^.

AEA and 2-AG are hydrolyzed by two major degradative enzymes, fatty acid amide hydrolase (FAAH) or monoacylglycerol lipase (MAGL), respectively^20^. Research shows that eCB degradation is involved in developing high alcohol preference^21^, anxiety, and excessive alcohol intake^22^, as well as in nociceptive signaling^23,24^. However, the effect of AUD on LHb eCB signaling, as well as the role of LHb eCB signaling on pain associated with AUD and alcohol consumption, is still unknown.

## Materials and methods

### Animals

Experiments were conducted on adult male Long-Evans rats (8-week old at the start of the experiments). All procedures were performed in accordance with the National Institute of Health’s guidelines and with the approval of the Animal Care and Utilization Committee of Rutgers, the State University of New Jersey. Rats were housed individually in ventilated Plexiglas cages in a climate-controlled room (20°C) under a 12-hour light/dark cycle (lights off at 11:00 A.M). Animals were allowed to acclimatize to the housing conditions and handling before the start of each experiment. Food and water were available ad libitum unless otherwise indicated.

### Chronic intermittent exposure to ethanol vapor or air

Alcohol dependence was induced using a chronic intermittent ethanol vapor exposure (CIE) protocol. Specifically, rats (2/cage) were placed in sealed chambers^25^ and exposed to ethanol vapor for 14 h/d as described^26^. Rats were maintained within a blood ethanol concentration (BEC) range of 150–250 mg/dl for 8–12 weeks. BECs were measured once per week and controlled at around 180 mg/dl. This BEC range is associated with mild-to-moderate withdrawal symptoms^27^. Behavioral experiments were conducted on rats exposed to CIE for 8–12 weeks (the exact number of weeks varied between cohorts to allow the emergence of stable pain hypersensitivity during withdrawal). Western blot and real-time PCR tests were conducted on tissue containing the LHb of rats exposed to CIE, which was harvested at ~24 h after their removal from the vapor chambers. Controls were intermittently exposed to ambient air for equal amounts of time.

### Measurement of ethanol concentration in vapor chamber air and animal blood

Ethanol vapor exposure was conducted in home-made chambers as described^25^. Ethanol concentration in the air of the vapor chambers was measured using a breathalyzer (Alco-Sensor III, Alcopro) as described^25^. BECs of rats were measured as described^28^ once per week during the CIE period. The blood sample collection and BEC measurement were as described^29^ (see “Supplementary Materials and Methods” for details).

### Measurement of voluntary ethanol consumption in the intermittent access to 20% ethanol 2-bottle free-choice (IA2BC) drinking paradigm

To assess the role of LHb eCB signaling on voluntary ethanol consumption, we trained a group of rats (*n* = 37) to drink alcohol. This group was subjected to alcohol vapor exposure for 8 weeks^30^ and then trained to drink ethanol using the IA2BC paradigm^31^.

### Operant ethanol self-administration

The self-administration test was conducted in a separate group (*n* = 20) of rats, which had been drinking ethanol in the IA2BC paradigm (as in “Measurement of voluntary ethanol consumption in the intermittent access to 20% ethanol 2-bottle free-choice (IA2BC) drinking paradigm”) for 4 weeks and had cannulas implanted in the LHb (see “Stereotaxic surgery and microinjection procedures”). The self-administration paradigm was as described^32^ (see “Supplementary Materials and Methods” for details).

### Stereotaxic surgery and microinjection procedures

The surgery and microinjection procedures were as described^11,32^. (see “Supplementary Materials and Methods” for details).

### Chemicals and application

All common salts were purchased from Sigma Aldrich (USA), including the selective MAGL inhibitor JZL184 (5 μg/200 nl/side), FAAH inhibitor URB597 (2 μg/200 nl/side), CB1 antagonist rimonabant (2 μg/200 nl/side), and WIN 55,212-2 (WIN, a specific CB1R agonist, 2 μg/200 nl/side). The doses of these chemicals were selected based on previous studies^22,33–36^. All chemicals were dissolved in a mixture of sterile aCSF and suspended in a 1:2:10 Tween 80, Dimethyl Sulfoxide (DMSO) solution following pilot work which confirmed this concentration allowed passage of high concentrations of this hydrophobic drug through the fine-gauged microinjectors.

### Western blot analysis, RNA isolation, and RT-qPCR analysis

Rats were perfused transcardially with ice-cold saline under deep anesthesia with sodium pentobarbital (50 mg/kg, i.p.). Brain tissue was sectioned with a vibratome into ice-cold artificial cerebral fluid (aCSF) containing (in mM): 126 NaCl, 2.5 KCl, 1.25 NaH_2_PO_4_, 1 MgCl_2_, 2 CaCl_2_, 25 NaHCO_3_, 1 l-ascorbate, and 11 glucose, and saturated with 95% O_2_/5% CO_2_ (carbogen). Tissue containing LHb was punched out from three to four 230 μm thick coronal slices on ice and was stored at −80 °C until processing. Western blot analysis was performed as described^32^ (see “Supplementary Materials and Methods for details”).

RNA isolation and RT-qPCR analysis were performed as previously described^12^. The tissue was stored at −80 °C until processing. Total RNA was extracted using the miRNeasy Mini Kit (Qiagen Inc., Germantown, MD, 217004). The quality of RNA was quantified using a spectrophotometer to ensure ratios of absorbance at 260–280 nm of 1.8–2.0. cDNA was synthesized using the QuantiTect^®^ Reverse Transcription kit (Qiagen Inc., Germantown, MD, 205313). Intron-spanning primers for mRNA were used to eliminate amplicons generated from any contaminating genomic DNA. Primer sequences are shown in Table 1. Quantitative PCR was performed on a CFX96 Touch™ Real-Time PCR Detection System (Bio-Rad Laboratories, Hercules, CA,1855196) using the QuantiTect SYBR Green PCR kit (Qiagen Inc., Germantown, MD, 204145) under the following conditions: 15 min at 95 °C followed by 39 cycles of 15 s at 94 °C, 30 s at 55 °C and 30 s at 70 °C. Real-time PCR was performed in duplicate for each sample. Ratios of mRNA expression of genes of interest to the endogenous control gene *Gapdh* were compared between groups.

**Table 1:** Primers sequences used for real-time polymerase chain reaction (RT-PCR).

Gene	Oligosense 5′→3′	Oligoantisense 5′→3′
*Cnr1*	ACCCATGGCTGAGGGTT	GGTACGGAAGGTGGTGTCTG
*Dagla*	GCAGTGTCAGGAGCAAGTCT	AGCAGCAACAGCTCTACAGG
*Daglb*	ACGACAAGGTGTACGAGCTG	TCCAGCTCTAGGTTCTCGCT
*Mgll*	CGGAACAAGTCGGAGGTTGA	AAGCATACCTTCACCCCTGC
*Napepld*	AGCTTATGAGCCAAGGTGGT	AGCTAAGGCAAAAGTCCCCC
*Faah*	GTTCACCTTGGACCCTACCG	AGAAGGGAATCAGCGTGTGG
*αCaMKII*	AGGATGAAGACACCAAAGTGC	GGTTCAAAGGC-TGTCATTCC
*βCaMKII*	ACCTCATTTGAGCCTGA-AGC	CAGGATAGTGGTGTGGATCG
*Gad67*	TCACAAGATGATGGGTGTGCTG	GTCATACTGCTTGTCTGGCTGGA
*Gad65*	TGACGCACTGCCAAACAACTC	TGCTGCTAATCCAACCATATCCAA

*Cnr1* cannabinoid receptor type 1, *Dagla/b* diacylglicerol lipase a/b, *Mgll* monoacylglicerollipase, *Napepld* N-acylphosphatidylethanolamine phospholipase D, *FAAH* fatty acid amide hydrolase, *α/βCaMKII* α/β form of calcium calmodulin-dependent protein kinase type II, *Gad67/65* Glutamate decarboxylase 67/65

### Immunofluorescence

The procedures involving brain fixation, immunofluorescence, and all antibodies used in this study were as described^28^ (see “Supplementary Materials and Methods” for details)

### Mechanical and thermal nociception tests

Pain sensitivity, including the paw withdrawal threshold (PWT) in response to mechanical stimuli (*von Frey hair test*) and paw withdrawal latency (PWL) to thermal stimuli (Hargreaves apparatus) was measured in a double-blinded manner. We first measured baselines before the rats were exposed to ethanol by taking the mean value of five continuous readings of the PWT or PWL. We then measured the PWT or PWL in rats subjected to 8 weeks of vapor exposure at 8 h after discontinuing the vapor. To overcome the possible effect of the increase in body weight on PWT and PWL, a group (*n* = 25) of age-matched ethanol-naïve rats served as controls at each time point.

The *von Frey hair test* was performed as described^37^. Briefly, the unrestrained rat was placed in a Plexiglas chamber on an elevated mesh screen in a test room. After at least 30 min acclimation, a series of von Frey hairs in log increments of force (0.41, 0.69, 1.20, 2.04, 3.63, 5.50, 8.51, 15.14 g) was applied perpendicularly to the plantar surface of the hind paw for 3 s. The 2.041-g stimulus was applied first. A sharp withdrawal of the hind paw indicated a positive response. If a positive response occurred, the next smaller von Frey hair was used; if a negative response was observed, the next larger von Frey hair was used. The test ended when (1) a negative response was obtained with the 15.14-g hair, and (2) 3 stimuli were applied after the first positive response. The PWT was determined by converting the pattern of positive and negative responses to the von Frey filament stimulation to a 50% threshold value with the formula provided by Dixon et al.^38^. The 50% PWT was determined by the formula Xf + kð, where Xf = last von Frey filament employed, k= Dixon value corresponding to response pattern, and ð = the mean difference between stimuli. The baseline PWT was similar to those reported for the ages of rats employed in this study^37^. The PWL to thermal stimuli was conducted as previously described^37^, using radiant heat (Hargreaves apparatus, Model 336 Analgesia Meter, IITC Life Science, Woodland Hills, CA) by aiming a beam of light from a lightbox through the glass plate at the middle of the plantar surface of the right and left hind paws. When the animal lifted its foot, the light beam was shut off, and the PWL was recorded. The PWL, measured in the unit of seconds, was defined as the length of time between the start of the light beam and the foot-lift. Each trial was repeated five times for each paw, while a 20-second cut-off time was used to avoid tissue damage. During the baseline test, rats showing a PWL above cut-off times were excluded from the study.

### Evaluation of physical signs of ethanol withdrawal

Physical signs of alcohol dependence usually manifest during acute withdrawal from chronic alcohol exposure and dissipate within the first 48 h after abstinence. To assess ethanol-induced physical dependence, we scored rats on a subjective rating scale as described^39^. Briefly, rats were gently transferred from the homecage to an isolated dimmed and silent (white noise no more than 55 dB) lightroom (illumination of 100–140 lux) at 8 h following removal from vapor chambers and were scored on a subjective rating scale from 0 to 2 for three prominent signs of withdrawal: (1) ventromedial distal limb flexion; (2) abnormal gait/posture; and (3) tail stiffness, as described^27,37^.

### Statistical analysis

All data were expressed as a mean ± SEM (standard error of the mean). Animal sample sizes chosen to ensure adequate statistical power were determined based on our preliminary studies. Animals were randomly assigned to different studies. Investigators were blinded to group allocations in the pharmacological behavioral experiments. Before the analysis, all data were checked for normality and homogeneity of variances. For gene and protein expression, the relative mRNA and protein expression of eCBs degradation enzymes and CB1Rs were analyzed using unpaired Student’s t-test between air and ethanol vapor-treated animals. Behavioral tests, including pain and alcohol consumption, between air and ethanol vapor-treated animals, were analyzed using a one- or two-way repeated-measures ANOVA, followed by Bonferroni or LSD post-hoc tests. Statistical significance was declared at *p* < 0.05.

## Results

### Chronic intermittent ethanol vapor exposure increases sensitivity to mechanical and thermal nociceptive stimuli

**Fig. 1:**
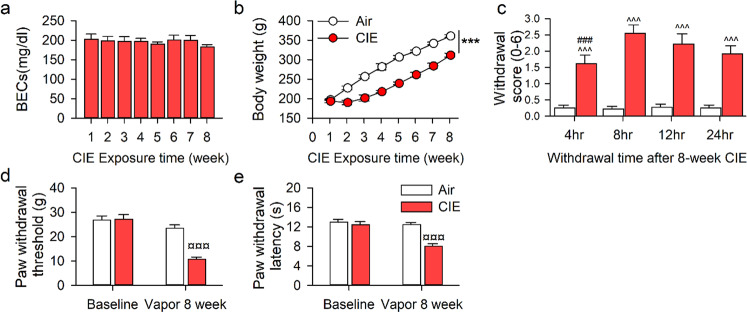
Pain hypersensitivity in rats withdrawing from chronic intermittent ethanol vapor exposure. Adult male Long-Evans rats were intermittently and randomly exposed to ethanol vapor (CIE, *n* = 18) or Air (AIR, *n* = 18) for 8 weeks. **a** The blood ethanol concentrations (BECs) were randomly collected from 11 of 18 CIE rats and measured once per week. **b** The body weight of rats during 8-week CIE or Air exposure (*n* = 11/group). **c** The physical withdrawal scores of CIE and AIR rats at 4, 8, 12, and 24 h abstinence after 8-week vapor exposure (*n* = 18/group). **d**, **e** The average paw withdrawal threshold or latency of rats before and after 8-week chronic vapor exposure (CIE *n* = 18, AIR *n* = 15). All data are expressed as mean ± SEM. ***p < 0.001, ^^^*p* < 0.001, ¤¤¤*p* < 0.001 vs. Air grou*p*, ###*p* < 0.001 vs 8–24 h withdrawal time within CIE group by two-way RM ANOVA.

We set up an alcohol-dependent rat model using a chronic intermittent ethanol exposure (CIE) protocol, as described^26,39^. To validate the CIE method, we measured ethanol concentrations in the air of vapor chambers and the blood of rats during multiple sessions. The vapor air ethanol concentration was constant at 4.5-5.0 g/dl throughout the experimental period. A one-way repeated-measures (RM) analysis of variance (ANOVA) assessing the BECs (mg/dl) of rats showed constant levels: 182.97 ± 13.68 in the 1st week maintained through 176.08 ± 6.55 in the 8th week of exposure (*F*_7 70_ = 0.14, *p* = 0.995, Fig. 1a). Before vapor exposure, the body weight of Air and CIE groups was not notably different (Air=205 ± 6 g; CIE = 201 ± 4 g). However, the CIE group gained significantly less weight than did the Air group during the vapor exposure period. A two-way RM ANOVA revealed a significant interaction on body weight between groups after 8 weeks of exposure (Air =426 ± 3 g, CIE = 375 ± 7 g, *F*_7 175_ = 64.17, *p* < 0.001, Fig. 1b).

To determine whether 8 weeks of CIE is sufficient to produce physical dependence, we rated the physical signs of animals at 4, 8, 12, and 24 h following cessation of vapor. A two-way RM ANOVA revealed a significant time × group interaction (*F*_3 143_ = 2.889, *p* = 0.04). The post-hoc test revealed significant withdrawal scores at all time points in CIE rats compared with the Air group (all *p* < 0.0001; Fig. 1c).

To determine whether nociceptive sensitivity had changed in these animals, we measured their PWL and PWT at 8 h following abstinence when their physical withdrawal scores were highest. A two-way RM ANOVA revealed a significant group × time interaction effect on PWL (*F*_1 59_ = 9.82, *p* = 0.007) and PWT (*F*_1 59_ = 58.16, *p* < 0.001). Post-hoc analyses revealed that CIE rats demonstrated hypersensitivity to mechanical and thermal stimuli compared to their pre-treatment baseline (*p* < 0.01), and to Air controls (*p* < 0.01, Fig. 1d, e).

### Effects of chronic alcohol exposure on endocannabinoid-related gene and protein expression in the LHb

**Fig. 2:**
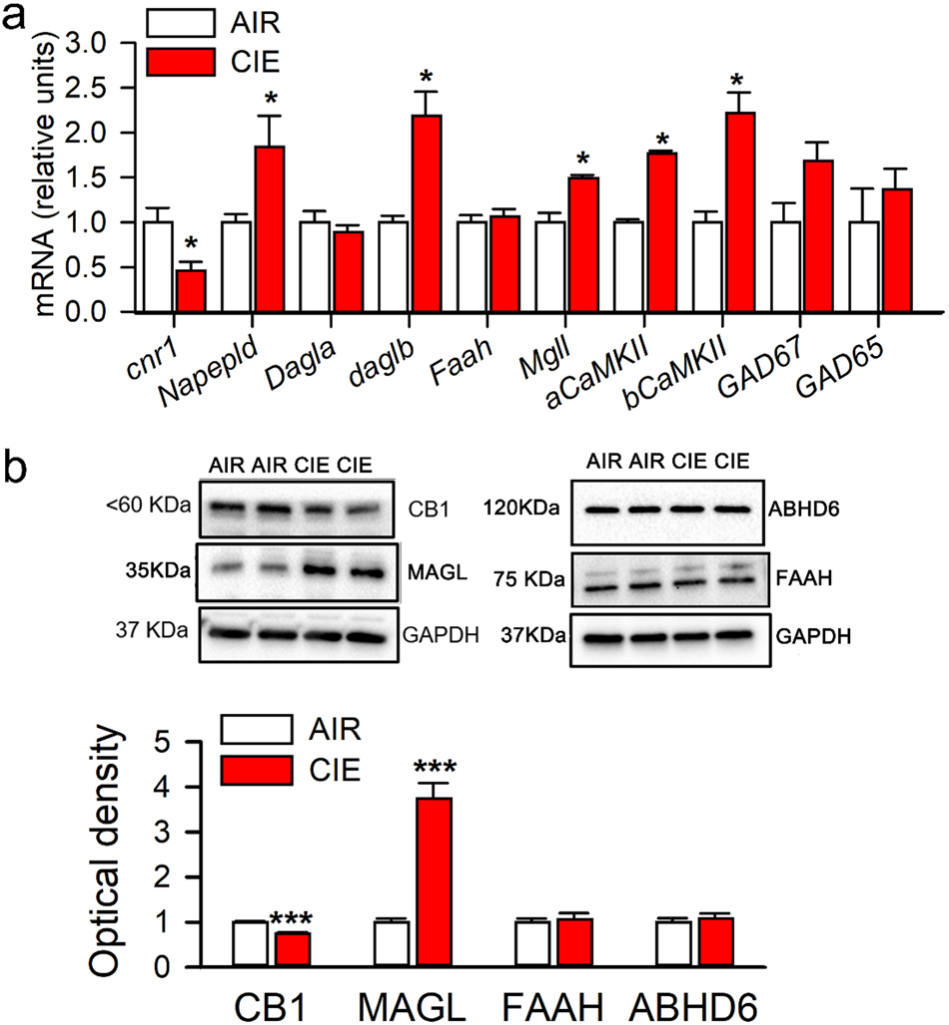
Effects of chronic intermittent ethanol exposure on endocannabinoid-related gene and protein expression in the LHb. Adult male Long-Evans rats were intermittently exposed to either ethanol vapor (CIE) or air (Air) for 8 weeks. The LHb tissue was harvested at 24 h after vapor off. The mRNA levels of genes associated with endocannabinoid signaling including *Cnr1, Napepld, Dagla, Daglb, Faah, Mgll*, as well as genes related to excitatory and inhibitory neurotransmission, like *aCaMKII, bCaMKII, Gad67*, and *Gad65* were measured in CIE (*n* = 11) and air control (Air, *n* = 6) rats. The protein levels of CB1Rs, MAGL, FAAH, and ABHD6 in the LHb were also measured. Panels (**a**) and (**b**) display quantitative mRNA levels and protein expression in these two groups. The Western blot and RT-qPCR tests were repeated three times. All data are expressed as mean ± SEM. *p < 0.05, ***, *p* < 0.001, CIE vs. Air group revealed by an unpaired *t* test.

To determine whether CIE alters levels of the enzymes responsible for eCB synthesis and degradation in the LHb, we measured the expression of genes involved in eCB signaling, as well as in excitatory and inhibitory neurotransmission. The mRNA level of *Cnr1* was decreased, whereas the mRNA levels of *Napepld, Daglb, Mgll, aCaMKII, and βCaMKII* but not *Gad65/67* were increased in CIE rats compared to the Air group (Fig. 2a). Also, the CB1R protein level was decreased, whereas the MAGL level was increased in CIE rats compared to the Air group. By contrast, neither FAAH nor ABHD6 levels of CIE rats were altered (Fig. 2b).

### LHb eCB breakdown inhibition or CB1R activation has an analgesic effect

Earlier research indicates that CB1R is expressed in the LHb^18,40^. Consistent with these findings, results obtained using confocal immunofluorescence assays showed that NeuN immunoreactive cells were positive for CB1R, MAGL, and FAAH proteins in the LHb of rats (Supplementary Fig. 1). We then measured PWT and PWL before and after bilateral intra-LHb infusion of the MAGL inhibitor JZL184, FAAH inhibitor URB597, or CB1R agonist WIN 55,212-2 (WIN). We also measured these after infusion of the CB1R antagonist rimonabant (RIM) alone, or in combination with the above compounds. The doses of inhibitors have previously been reported to effectively raise brain eCB levels^41^.

**Fig. 3:**
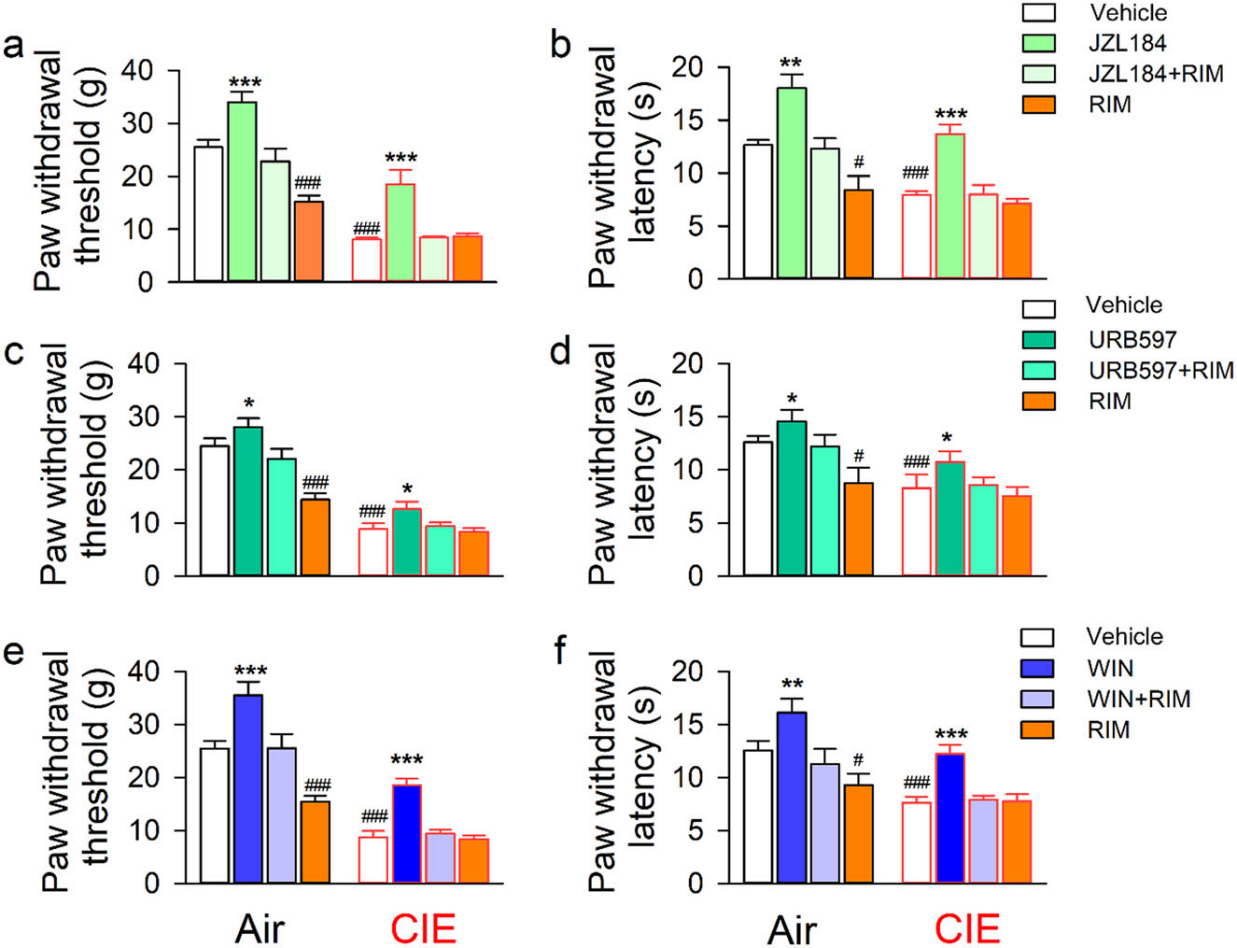
Inhibiting LHb MAGL or FAAH or activating CB1R has an analgesic effect in both CIE and air-exposed rats. Adult male Long-Evans rats underwent CIE or air exposure for 8 weeks. At 24 h abstinence from the last vapor exposure, these rats randomly received bilateral intra-LHb infusion of JZL184 (MAGL inhibitor, *n* = 13 rats/ group), URB597 (FAAH inhibitor, *n* = 14 rats/group), or WIN 55,212-2 (CB1R agonist, *n* = 14 rats/group) alone or combined with rimonabant (RIM, an inverse CB1R agonist) 30 min before the behavioral test. The bar graphs summarize the effect of these compounds on the paw withdrawal threshold (PWT, **a**, **c**, and **e**) or latency (PWL, **b**, **d**, and **f**) in all subjects tested. All data are expressed as mean ± SEM. ^###^*p* < 0.001 vs vehicle or Air group; ^*^p < 0.05, ^**^p < 0.01, ^***^p < 0.001 vs vehicle, JZL + RIM or RIM within each vapor group, revealed by two-way RM ANOVA followed by Bonferroni post-hoc test.

Measuring the effect of JZL184, a significant vapor × treatment effect on PWT (*F*_3 103_ = 14.74, *p* < 0.001) and on PWL (Vapor, *F*_1 103_ = 46.52, *p* < 0.001; Treatment, *F*_3 103_ = 18.38, *p* < 0.001) was revealed by two-way RM ANOVA. Specifically, for the CIE group, JZL184 attenuated hypersensitivity to mechanical and thermal stimuli (all *p* < 0.05), but this was not seen either in JZL184 with RIM, or RIM alone, as compared to vehicle. Conversely, for the Air group, JZL184 attenuated sensitivity to mechanical and thermal stimuli (all *p* < 0.05), whereas RIM increased them, as compared to vehicle (Fig. 3a, b).

As illustrated in Fig. 3c–f, both URB597 and WIN had an analgesic effect, which was blocked by RIM, in both the CIE and Air groups. Two-way RM ANOVA detected a significant vapor × treatment interaction on both URB597 (PWT, *F*_3 103_ = 17.70, *p* < 0.001; PWL, *F*_3 103_ = 13.88, *p* < 0.001) and WIN tests (PWT, *F*_3 103_ = 9.26, *p* < 0.001; PWL, *F*_3 103_ = 10.07, *p* < 0.001).

### LHb eCB metabolism inhibition or CB1R activation reduces ethanol consumption in ethanol-dependent rats

**Fig. 4:**
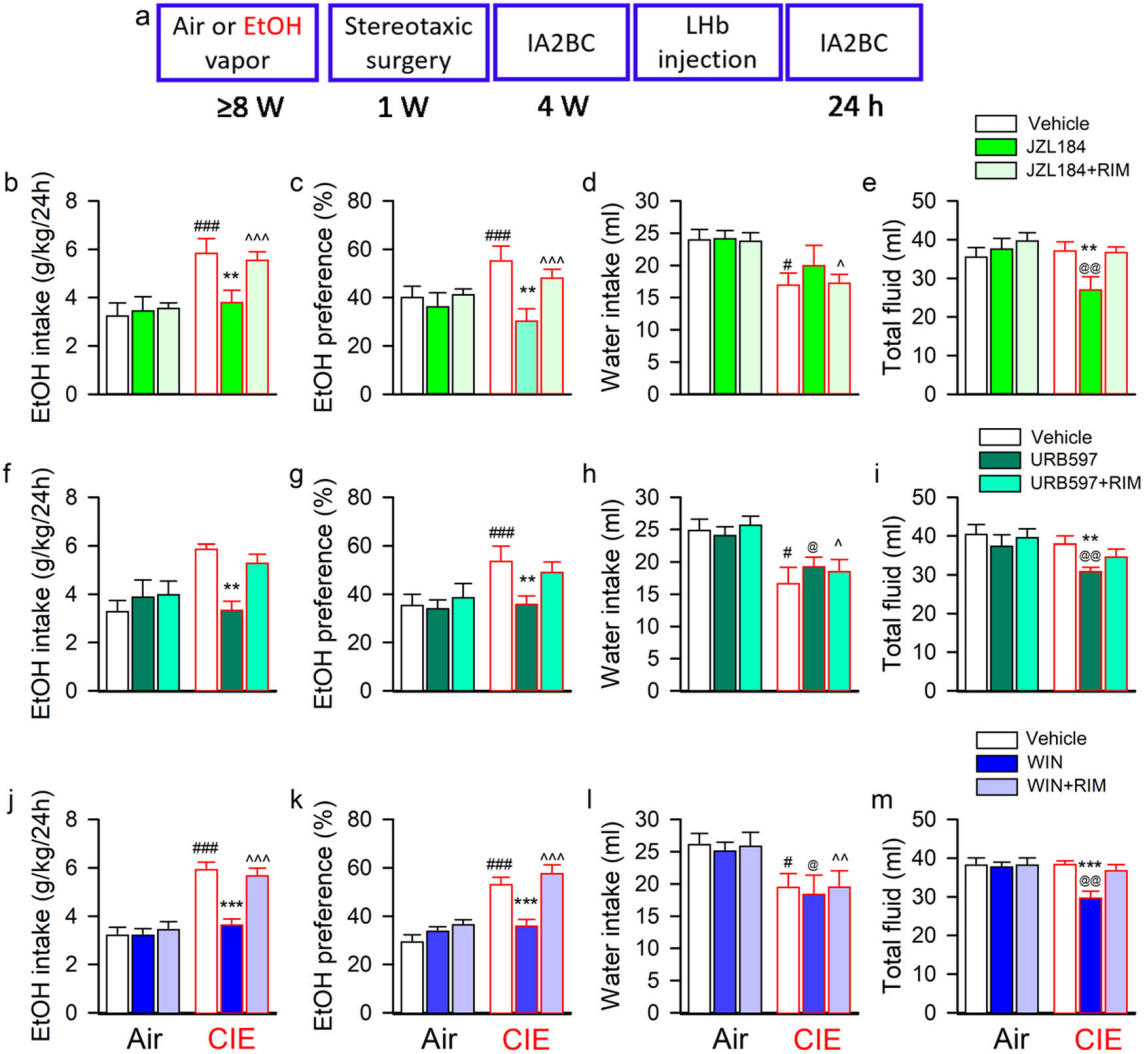
LHb eCB-degrading enzyme inhibition or CB1 receptor activation decreases voluntary alcohol consumption in rats with a chronic intermittent ethanol vapor exposure history. Panel (**a**) is the schematic diagram showing the experimental timeline. Adult male Long-Evans rats were intermittently exposed to either ethanol vapor (CIE) or air for 8 weeks, followed by voluntary drinking in the intermittent access to 20% ethanol 2-bottle free-choice (IA2BC) paradigm for 4 weeks. At 24-h abstinence from the last drinking session, JZL184 (5 μg/200 nl/side; **b–e**), URB597 (4 μg/200 nl/side; **f–i**) or WIN 55,212-2 (WIN, 4 μg/200 nl/side; **i**–**k** and **m**) alone or with rimonabant (RIM, 2 μg/200 nl/side), was bilaterally injected into the LHb 30 min before the beginning of a drinking session. The effect of LHb administration of these compounds on ethanol consumption was summarized in panels b-m. All data are expressed as mean ± SEM, *n* = 11–12/group for each compound. **p < 0.01, ***p < 0.001 vs Vehicle or JZL184/URB597/WIN + RIM within group; ^#^*p* < 0.05, ^##^*p* < 0.01, ^###^*p* < 0.001 vs same treatment among distinct grou*p*s, revealed by two-way RM ANOVA followed by Bonferroni post-hoc test.

To assess whether LHb eCB signaling affects ethanol consumption, we trained CIE and Air rats to drink alcohol in the homecage under the IA2BC paradigm (Method “Measurement of voluntary ethanol consumption in the intermittent access to 20% ethanol 2-bottle free-choice (IA2BC) drinking paradigm”, Fig. 4a). We first investigated the effect of CIE on ethanol consumption. Two-way RM ANOVA revealed a significant effect of vapor exposure history on ethanol consumption during the 4 weeks (*F*_1 383_ = 24.325, *p* < 0.0001, Supplementary Fig. 2a). Ethanol intake (g/kg) of CIE rats was elevated from 2.4 ± 0.3 g/kg/24 h in the first drinking session to a stable level of 5.5 ± 0.4 g/kg/24 h by the end of the 12th drinking session. CIE rats drank significantly more ethanol than Air rats by the 10–12th sessions (all *p* < 0.05).

Thereafter, we divided the CIE and air rats into three subgroups to examine the effect on voluntary alcohol consumption of intra-LHb JZL184, URB597, or WIN alone or in combination with RIM. All the chemicals were injected bilaterally into the LHb of rats at 24 h withdrawal, 30 min before the beginning of an alcohol drinking session.

For JZL184, two-way RM ANOVA revealed a significant treatment × vapor interaction on intake (*F*_2 71_ = 3.691, *p* = 0.041) and preference for ethanol (*F*_2 71_ = 4.368, *p* = 0.025). Post-hoc analysis revealed that, as compared to vehicle, JZL184 alone significantly reduced ethanol intake, preference, and total fluid intake in CIE rats (all *p* < 0.05, Fig. 4b–e), which were all blocked by RIM. Nevertheless, JZL184 did not significantly alter the ethanol drinking behavior in the Air group (all *p* > 0.05).

For URB597, two-way RM ANOVA revealed a significant treatment × vapor interaction on intake (*F*_2 71_ = 10.22, *p* < 0.001) and preference for ethanol (*F*_2 71_ = 18.77, *p* < 0.001), as well as on total fluid intake (*F*_2 71_ = 5.18, *p* = 0.014). Post-hoc analysis found that URB597 also significantly decreased ethanol intake, preference, and total fluid intake (all *p* < 0.05, Fig. 4f–i), without altered water intake, as compared to vehicle. The effect of URB597 was reversed by blocking CB1R with RIM. Again, URB597 did not alter ethanol consumption in the Air group (all *p* > 0.05).

Additionally, we evaluated whether LHb CB1R activation changes ethanol drinking behavior and whether it is related to the state of alcohol dependence. Two-way RM ANOVA revealed a significant treatment × vapor interaction on intake (*F*_2 71_ = 17.57, *p* < 0.001) and preference for ethanol (*F*_2 71_ = 11.49, *p* < 0.001), as well as on water intake (*F*_2 71_ = 3.78, *p* = 0.039). Post-hoc analysis revealed that intra-LHb injection of WIN significantly decreased ethanol intake, preference, and total fluid intake, without changing water intake, as compared to a vehicle infusion (all *p* < 0.05, Fig. 4j–m). The effect of WIN was antagonized by RIM. WIN did not significantly alter ethanol consumption in the Air group (all *p* > 0.05). Moreover, RIM alone did not alter ethanol consumption in either CIE or Air groups (Supplementary Fig. 2b–e).

### Inhibition of LHb MAGL or activation of LHb CB1 reduce ethanol seeking behavior in CIE rats

**Fig. 5:**
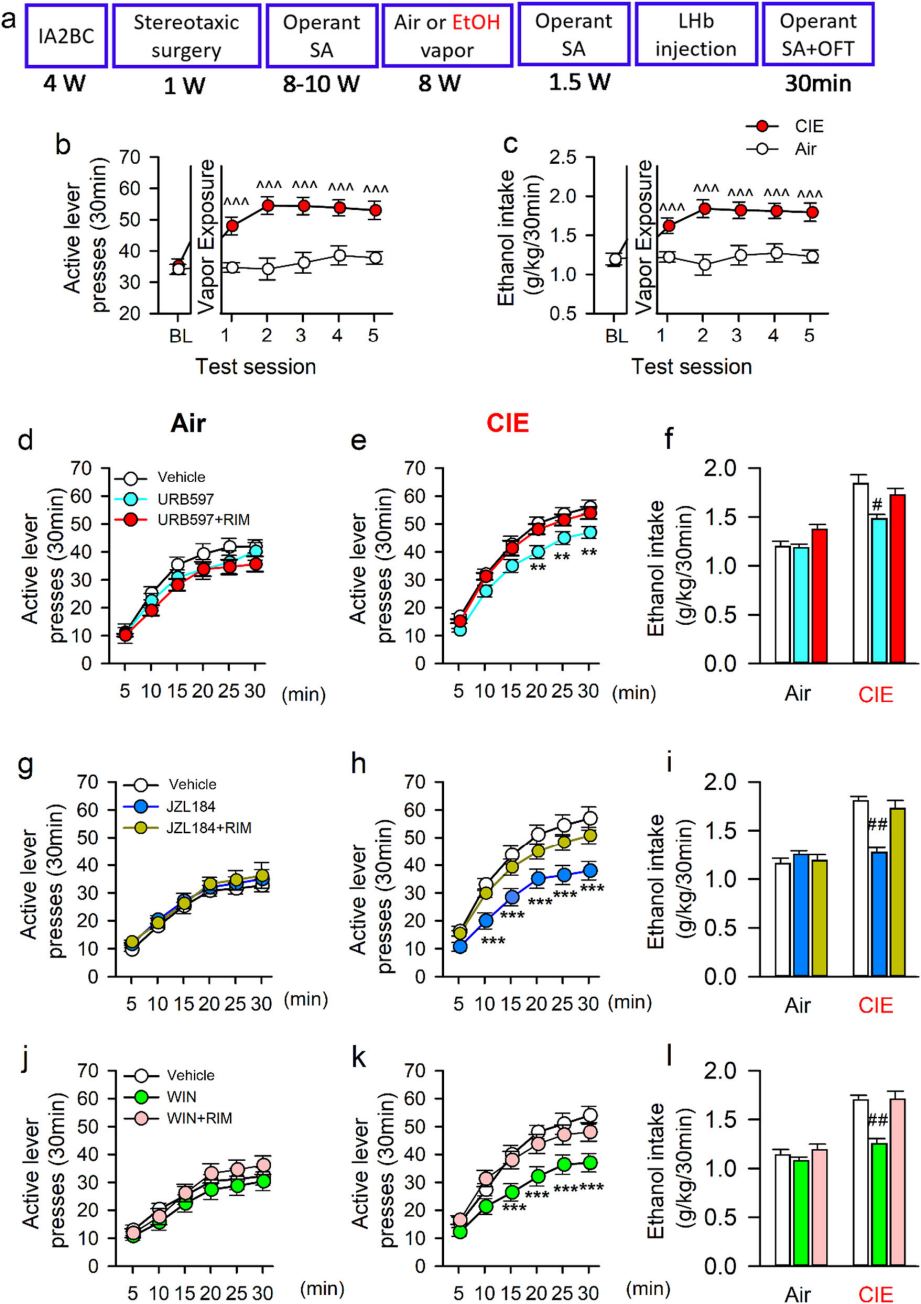
Inhibiting LHb eCB hydrolysis or activating LHb CB1Rs decreases alcohol-seeking in rats with a history of chronic intermittent exposure to ethanol vapor (CIE). Panel (**a**) depicts the experimental timeline. Adult male Long-Evans rats were first trained to drink alcohol for 4 weeks in the intermittent access 2-bottle free-choice (IA2BC) paradigm, followed by training for ethanol self-administration (SA) in the operant chambers in the FR1 paradigm for 8–10 weeks. They then were exposed to either CIE or Air vapor for 8 weeks. The number of active lever presses (**b**) and ethanol consumption (**c**) of CIE exposed rats significantly increased compared to before CIE or Air control rats. At 24-h abstinence from the last drinking session, a selective FAAH inhibitor (URB597), MAGL inhibitor (JZL184), CB1R agonist WIN55,212-2 (WIN), or Vehicle was bilaterally injected into the LHb 30 min before a drinking session. Panel (**d**–**l**) summarizes the effect of these compounds on active lever presses in both Air (**d**, **g**, and **j**) and CIE rats (**e**, **h**, and **k**), as well as the change of ethanol consumption (**f**, **l**). All data are expressed as mean ± SEM. ^^^*p* < 0.001 vs baseline (BL) within CIE or Air group revealed by one-way RM ANOVA, ***p < 0.001 vs Vehicle or URB597/JZL184/WIN + RIM within CIE group revealed by two-way ANOVA; ^#^*p* < 0.05 vs Vehicle or URB597/JZL184/WIN + RIM within CIE group, by one-way ANOVA followed by Bonferroni post-hoc test. *n* = 16-18 rats/group.

Next, we assessed the role of LHb eCB signaling in alcohol-seeking behaviors. As illustrated in Fig. 5a, after 4 weeks of drinking in the homecages under the IA2BC paradigm, rats were trained to self-administer (SA) ethanol in the operant chambers using an FR1 program until they developed a stable operant response (35 ± 2) and ethanol intake (1.18 ± 0.08 g/kg/30 min) during 20 conditioning training sessions. These rats were then exposed to CIE or Air vapor for 8 weeks. After that, they were brought back to the operant chambers for five sessions to re-establish the baseline (mean value of five consecutive sessions) of operant ethanol response. Two-way RM ANOVA revealed a significant vapor × time interaction on the number of active lever presses (*F*_5 215_ = 6.762, *p* < 0.001) and ethanol intake (*F*_5 215_ = 7.972, *p* < 0.001). The Air group maintained alcohol-seeking motivation and consumption levels unchanged from before vapor exposure. CIE rats, on the other hand, demonstrated much higher active lever presses (53 ± 3) and ethanol intake (1.82 ± 0.12 g/kg/30 min) than their Air counterparts (Fig. 5b, c). This is consistent with the previous finding that CIE enhances ethanol operant self-administration behavior^42,43^.

These rats were then randomly divided into three subgroups testing the effect of local administration of URB597, JZL184, and WIN, alone or combined with RIM on SA. For URB597, one-way RM ANOVA revealed a significant drug treatment × time interaction effect on active lever presses in CIE (*F*_10 359_ = 9.004, *p* < 0.001) but not Air-exposed rats (*F*_10 323_ = 303.73, *p* > 0.05). Compared to vehicle, URB597 reduced the number of active lever presses in CIE rats, which occurred at 20-30 min during the test (all *p* < 0.05), an effect inhibited by RIM (Fig. 5d–f).

Similarly, JZL184 had a significant treatment × time effect on the active lever presses in the CIE (*F*_10 359_ = 8.285, *p* < 0.007) but not the Air group (*F*_10 323_ = 304.81, *p* > 0.05). Notably, within the CIE group, JZL184 significantly decreased the number of active lever presses as compared to the vehicle (all *p* < 0.05), an effect partially blocked by RIM (Fig. 5g–i).

Moreover, LHb CB1R activation also significantly suppressed alcohol-seeking behavior, with a significant treatment × time effect on active lever presses in the CIE (*F*_10 359_ = 5.352, *p* < 0.001) but not the Air group (*F*_10 323_ = 195.93, *p* > 0.05). Among CIE rats, compared to vehicle, WIN reduced active lever presses (all *p* < 0.05), an effect again reversed by RIM (Fig. 5j–l).

Consistently, the reduction in active lever press by inactivating LHb eCB-degrading enzymes or activating CB1Rs also resulted in lower ethanol consumption of CIE rats (all *p* < 0.05). Also, RIM alone did not affect the active lever presses in rats regardless of their vapor exposure history (Supplementary Fig. 3).

Although intra-LHb injection of URB597, JZL184, or WIN increased locomotor activity of both Air and CIE rats (Supplementary Fig. 4, *F*_3__39_ = 5.029, *p* = 0.005), these manipulations did not alter the inactive lever presses as compared to vehicle (data not shown).

## Discussion

We report here that the eCB system is impaired in the LHb of alcohol-dependent rats. These animals displayed hypersensitivity to mechanical and thermal nociceptive stimuli and elevated voluntary ethanol consumption. Pharmacological corrections of the impaired LHb eCB signaling effectively reduced pain and ethanol intake. These findings highlight the vital role of the LHb eCB system in pain associated with AUD.

AUD is typically accompanied by the emergence of negative emotional states that constitute a motivational withdrawal syndrome when access to alcohol is disrupted^44,45^. Research has shown that withdrawal from alcohol dependence is associated with long-lasting pain^37,46–50^. Here we reported that rats displayed hypersensitivity to nociceptive stimuli during acute withdrawal from chronic ethanol vapor exposure. Our findings are consistent with previous reports^26,51–53^.

Notably, in the LHb of animals acutely withdrawing from chronic intermittent ethanol exposure, the mRNA expression of *aCaMKII* and *βCaMKII* but not *Gad65/67* was increased, suggesting enhanced neuronal activation^19,54^. This is consistent with the observation that LHb neurons are activated during withdrawal from chronic ethanol administration^12,32,55^.

The eCBs are potent regulators of synaptic function throughout the central nervous system, mainly as retrograde messengers suppressing transmitter release (both transiently and in a long-lasting manner), at both excitatory and inhibitory synapses^56,57^. Given that increased glutamatergic transmission is a major factor contributing to LHb neuron hyperactivity in animals withdrawing from chronic alcohol exposure^11,12^, and that CB1Rs are present at presynaptic terminals^18^, diminished eCB signaling may contribute to the increased presynaptic glutamate release in the LHb of rats withdrawing from chronic ethanol.

AUD-induced impairment of the eCBs has been reported previously. A reduction of eCB levels was found in the midbrain of animals subjected to chronic alcohol intoxication^58^. Short-term alcohol exposure also diminishes AEA level in the hypothalamus, amygdala and caudate-putamen, and also reduces 2-AG levels in the prefrontal cortex^59^. The reduction of eCB levels following chronic ethanol exposure may arise from reduced synthesis and/or enhanced inactivation mechanisms. Increased levels of NAPE-PLD, DAGLα/β, AEA and 2-AG primary synthetic enzymes may facilitate eCB synthesis, whereas increased levels of MAGL and ABHD6 (α, β hydrolase domain 6) may enhance 2-AG hydrolysis and clearance^60–62^. This possibility is supported by a study showing that MAGL mRNA expression was increased post-mortem in the prefrontal cortex of alcoholics^15^. In keeping with this finding, we report here that in the LHb of rats withdrawing from chronic ethanol exposure, MAGL levels were increased. Also, mRNA levels of both *Napepld* and *Daglb* were elevated. These results suggest that chronic alcohol administration and withdrawal alters LHb eCB biosynthesis and degradation, especially through MAGL, an important molecule for 2-AG hydrolysis.

Previous studies have found that CB1R expression in several subcortical structures was downregulated either immediately after chronic ethanol administrations or during acute withdrawal^59,63–66^, and that chronic heavy drinking leads to reduced CB1R availability, especially in the ventral striatum and mesotemporal lobe^15^. In keeping with these findings, we reported that CB1R protein levels were reduced in the LHb of rats withdrawing from chronic ethanol exposure, suggesting that a reduction in CB1 signaling is one of the adaptations in the LHb during the development of alcohol dependence. CB1R down-regulation may counteract the adaptation at GABAergic and glutamatergic synapses after chronic ethanol consumption^67^. Although we observed that increases in *aCaMKII* and *βCaMKII* levels were accompanied by a decrease in CB1R expression, it remains to be determined whether this reduction in CB1Rs plays a causal role in the enhanced glutamatergic transmission to and the hyperactivity of the LHb neurons in rats withdrawing from chronic alcohol.

We observed that intra-LHb injection of the MAGL inhibitor JZL184, the FAAH inhibitor URB597, or the CB1R agonist WIN55,212-2 produced an analgesic effect, regardless of ethanol or air exposure history, implying that alcohol exposure does not change eCB pain responses. Remarkably, blocking the activity of eCB hydrolysis enzymes in the LHb significantly reduces pain hypersensitivity, accompanied by decreased voluntary alcohol consumption. Specifically, intra-LHb JZL184, more so than URB597 treatment, attenuated alcohol consumption, implying a critical and potent role of LHb eCBs in suppressing alcohol consummatory behaviors. Our findings are consistent with prior studies showing that 2-AG levels in the basal lateral amygdala are decreased during alcohol withdrawal and that systemic administration of MAGL inhibitors ameliorates affective disturbances and reduces alcohol consumption^66,68,69^. Besides, our data showing that intra-LHb injection of CB1R agonist WIN55212-2 reduced ethanol consumption, whereas LHb rimonabant administration did not change ethanol consumption in homecages or operant chambers, further support the critical role of LHb eCBs/CB1Rs in the regulation of ethanol consumption. Notably, rimonabant attenuated pain in alcohol-naïve rats, but not in the CIE rats. This could be due to a floor effect following CIE that prevents any further effect of rimonabant, or maybe the eCB system is impaired such that CB1 activation no longer occurs.

By contrast, it has been widely reported that an enhanced ethanol-reinforcing effect or high drinking amount is linked with elevated brain eCB transmission or decreased eCB hydrolysis enzyme protein expression and activity^70–72^. Moreover, enhancing CB1R agonism generally increases alcohol consumption, whereas systemic administration^73–75^ or VTA or NAc^76^ microinfusion of rimonabant reduces voluntary ethanol consumption and seeking behaviors, indicating the eCBs mediates ethanol reinforcement through the functional modulation of the mesocorticolimbic dopaminergic pathway^58,63,77^. These studies also support CB1R and FAAH as therapeutic targets in the treatment of excessive alcohol consumption^78,79^.

Generally, we agree with the above view but propose that inconsistencies regarding the effect of the eCB system in AUD may be due to the widely varied distribution of eCBs and receptors within complex neural circuits, or to the heterogeneity of neurons in these brain regions. Even CB1R agonists at different doses applied in the same region may cause distinct behavioral phenotypes. For example, CB1R agonism was reported to have a biphasic effect in regulating alcohol consumption within the VTA. Linsenbardt et al. reported that WIN 55,212-2 at a lower dose (0.5 mg/kg) increased ethanol intake, whereas at higher doses (1–2 mg/kg) it decreased ethanol intake. More interestingly, intra-pVTA WIN 55,212-2 injections both increased (0.25 and 0.50 µg/side) and decreased (2.50 µg/side) ethanol intake^36^. Therefore, considering the LHb participates in anti-reward circuits^80^ and functionally controls VTA DA neuron activity in a direct or indirect manner, this would explain why the effect of CB1R pharmacological manipulation in the LHb differs from that in other brain areas, such as the VTA.

Chronic vapor exposure did not significantly alter FAAH levels, and its pharmacological inactivation suppressed alcohol consumption. This could be due to FAAH inhibition enhancing eCBs signaling and restoring CB1 activity. These data also suggest that cannabis and alcohol may interact and that cannabinoids may act either as substitutes for alcohol by counteracting withdrawal symptoms or as therapeutic agents to help in alcohol cessation. Overall, our findings reported here suggest a dysregulation of CB1Rs and eCBs in AUDs.

The relationship between alcohol and pain is complex. Acute alcohol can reduce pain, but chronic alcohol abuse can aggravate pain. Withdrawal from chronic excessive alcohol consumption can lead to the emotional misery of hyperkatifeia, including pain^81^. Recently, an increasing number of in vivo studies, including ours, have highlighted the role of the LHb in pain processing. For example, aberrant LHb-DNR (dorsal nucleus of raphe) circuit function, resulting in a decreased DRN serotonin levels, is linked to neuropathic pain^82^. GPR139, a member of the orphan G protein-coupled receptor superfamily in the LHb, may play a role in alcohol dependence and its related hyperalgesia^52^. We previously reported that the downregulated M-type potassium channels and elevated type 1 TRPV receptors contribute to the hyperactivity of LHb neurons and cause nociceptive hypersensitivity during alcohol withdrawal^10,12,55^. Our current study shows that inhibiting LHb eCB degradation or activating LHb CB1Rs reduces nociceptive hypersensitivity in alcohol-dependent animals. Research shows that eCBs have anti-nociceptive effects under physiological and pathological conditions at both spinal and supraspinal levels, such as the periaqueductal gray, thalamus, rostral ventromedial medulla, and amygdala^83^. Here, we reported that inactivating MAGL, the primary 2-AG catabolic enzyme, reduces hypersensitivity to thermal and mechanical stimuli, in line with a previous report showing systemic administration of JZL184 attenuates inflammatory pain^84^. A recent study reported that eCB signaling is required for LTD in the LHb, and that stress selectively impairs presynaptic LTD through interference with 2-AG synthesis^19^. Therefore, one plausible interpretation for the alleviated pain hypersensitivity is that either pharmacological activation of CB1Rs or enhanced 2-AG levels (by inhibiting its hydrolysis) might sufficiently restore presynaptic LTD, suppressing the hyperactive LHb neuronal output. Future studies are needed to test this possibility.

In summary, we report that alcohol withdrawal decreases CB1R levels and enhances MAGL mRNA levels in the LHb. Inhibition of LHb MAGL or FAAH attenuates hyperalgesia and ethanol consumption. Thus, our findings suggest that LHb eCBs are a vital modulator of alcohol consumption and hyperalgesia associated with AUD.

## Supplementary information

Supplementary figure legends

Supplementary Fig. 1

Supplementary Fig. 2

Supplementary Fig. 3

Supplementary Fig. 4
