# Supplementary material for: Endocannabinoid signaling in the lateral habenula regulates pain and alcohol consumption
Source: Transl Psychiatry. 2021 Apr 14;11:220. doi: 10.1038/s41398-021-01337-3 (PMC8046806; doi:10.1038/s41398-021-01337-3)
Supplement: Supplementary file 1 — Supplementary figure legends [file 41398_2021_1337_MOESM1_ESM.docx]

**Supplementary Figure 1.**

**MAGL, FAAH, and CB1R are expressed in the LHb neurons.**

The representative confocal micrograph demonstrates the co-localization of MAGL, FAAH, and CB1R (red) with NeuN immunoreactive cells (green) in the LHb of naïve Long-Evans rats. Scale bar=20 μm.

**Supplementary Figure 2.**

**Intra-LHb rimonabant does not significantly influence the voluntary ethanol consumption of rats with a chronic intermittent ethanol (CIE) vapor exposure history.**Adult male Long-Evans rats were subjected to an 8-weeks intermittent ethanol (EtOH) or air vapor exposure and were then transferred to drink ethanol under the intermittent access 2-bottle free choice (IA2BC) paradigm for 4 weeks. Panel **a** shows CIE exposure significantly elevated ethanol intake.  **p*<0.05 vs. AIR, by two-way RM ANOVA, n=16 rats/group; at 24-h abstinence from the last drinking session, rimonabant (RIM, 2μg/200nl/side) was bilaterally injected into the LHb 30 min before a drinking session. There are significant vapor effects on ethanol intake (F_1 47_=28.911, *p*<0.001), ethanol preference (F_1 47_=52.95, *p*<0.001) and water intake (F_1 47_=33.47, *p*<0.001). Panels **b-e** summarize the effects of RIM on ethanol consumption behavior. All data are expressed as mean ± SEM. **p*<0.05 vs. AIR, by two-way RM ANOVA followed by Bonferroni post hoc test, n=12 rats/vapor group.

**Supplementary Figure 3.**

**Intra-LHb rimonabant does not significantly affect operant self-administration in rats with a chronic intermittent ethanol (CIE) vapor exposure history.**

Adult male Long-Evans rats were first trained to drink alcohol for 4 weeks in the intermittent access 2-bottle free choice (IA2BC) paradigm, followed by training in ethanol self-administration (SA) in the operant chambers in the FR1 paradigm for 8-10 weeks. They then were exposed to either CIE or Air vapor for 8 weeks and then continued to self-administer 20% ethanol under a FR1 paradigm. Panels **a-c** summarizes the effects of intra-LHb vehicle or the CB1 receptor rimonabant (RIM) on active lever presses and ethanol consumption in rats with Air and CIE exposure history. All data are expressed as mean ± SEM. **p*<0.05 vs. Air, by two-way RM ANOVA, n=15 rats/group.

**Supplementary Figure 4.**

**LHb eCB-degrading enzyme inhibition or CB1 receptor activation increases locomotion.**

At 24h abstinence from operant ethanol self-administration, rats with an Air or CIE vapor exposure history received bilateral intra-LHb infusion of selective FAAH inhibitor (URB597), MAGL inhibitor (JZL184), CB1R agonist WIN55,212-2 (WIN), or vehicle for 30 min. The rats were then placed in the center of an open arena (60×60×40 cm; 5.5 lux) and tracked over 10 minutes. The graphs show the total distance traveled after each chemical administration. All data are expressed as mean ± SEM. #*p*<0.05 vs vehicle within Air or CIE group, by two-way RM ANOVA, n=15 rats/group.
