# Supplementary figures and images for: Endocannabinoid signaling in the lateral habenula regulates pain and alcohol consumption
Source: Transl Psychiatry. 2021 Apr 14;11:220. doi: 10.1038/s41398-021-01337-3 (PMC8046806; doi:10.1038/s41398-021-01337-3)

Supplementary Fig.1

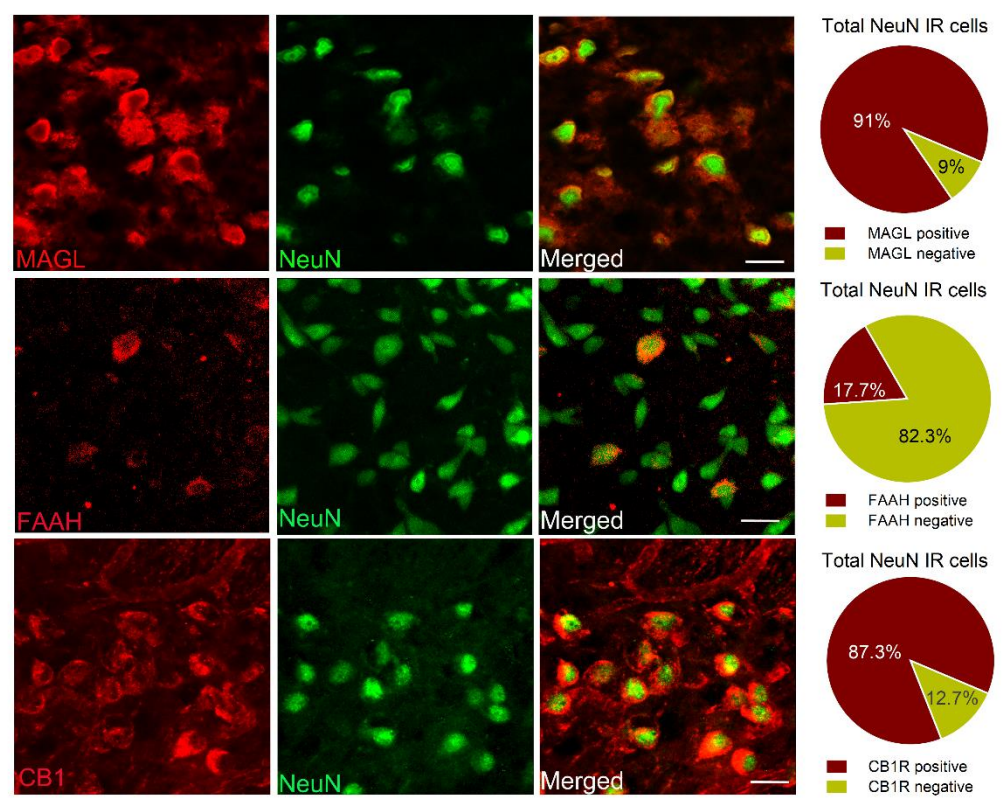

Supplement: Supplementary file 2 — Supplementary Fig. 1 [file 41398_2021_1337_MOESM2_ESM.pdf]

Supplementary Figure 1

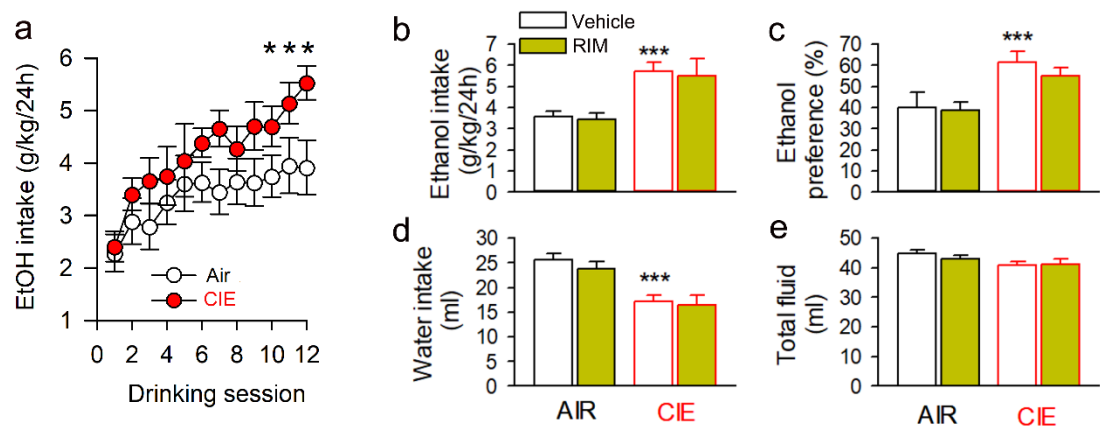

Supplement: Supplementary file 3 — Supplementary Fig. 2 [file 41398_2021_1337_MOESM3_ESM.pdf]

Supplementary Figure 2

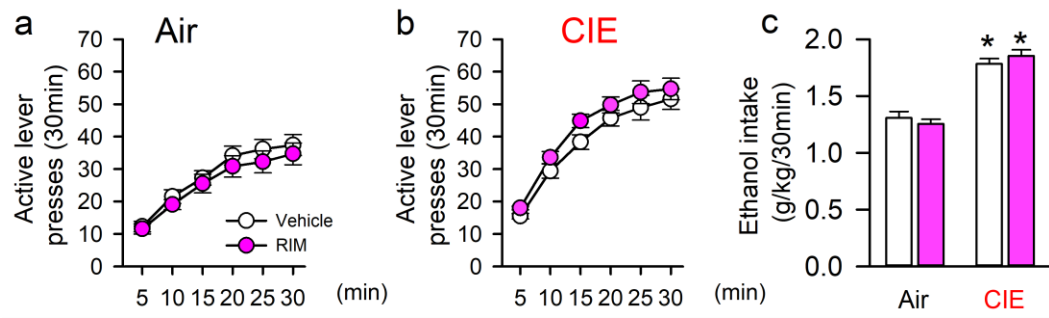

Supplement: Supplementary file 4 — Supplementary Fig. 3 [file 41398_2021_1337_MOESM4_ESM.pdf]

Supplementary Figure 3

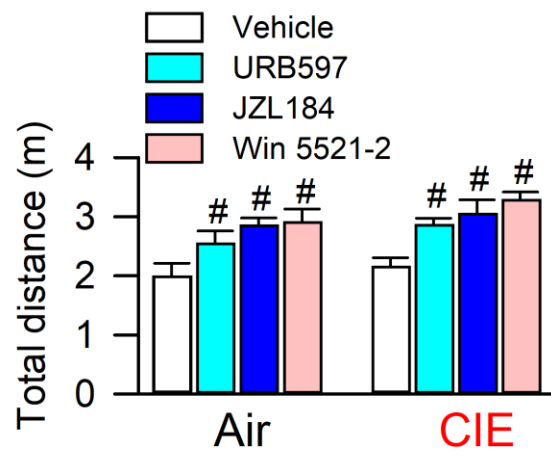

Supplement: Supplementary file 5 — Supplementary Fig. 4 [file 41398_2021_1337_MOESM5_ESM.pdf]
